# Lipopolysaccharide enhances TGF‐β1 signalling pathway and rat pancreatic fibrosis

**DOI:** 10.1111/jcmm.13526

**Published:** 2018-02-09

**Authors:** Li Sun, Ming Xiu, Shuhua Wang, David R. Brigstock, Hongyan Li, Limei Qu, Runping Gao

**Affiliations:** ^1^ Department of Hepatic Biliary Pancreatic Medicine First Hospital of Jilin University Changchun China; ^2^ Department of Surgical Gastroenterology First Hospital of Jilin University Changchun China; ^3^ The Research Institute at Nationwide Children's Hospital Columbus OH USA

**Keywords:** alcohol, lipopolysaccharide, pancreatic fibrosis, pancreatic stellate cell, TGF‐β1

## Abstract

Pancreatic stellate cells (PSCs) play a critical role in fibrogenesis during alcoholic chronic pancreatitis (ACP). Transforming growth factor‐beta1 (TGF‐β1) is a key regulator of extracellular matrix production and PSC activation. Endotoxin lipopolysaccharide (LPS) has been recognized as a trigger factor in the pathogenesis of ACP. This study aimed to investigate the mechanisms by which LPS modulates TGF‐β1 signalling and pancreatic fibrosis. Sprague‐Dawley rats fed with a Lieber‐DeCarli alcohol (ALC) liquid diet for 10 weeks with or without LPS challenge during the last 3 weeks. *In vitro* studies were performed using rat macrophages (Mφs) and PSCs (RP‐2 cell line). The results showed that repeated LPS challenge resulted in significantly more collagen production and PSC activation compared to rats fed with ALC alone. LPS administration caused overexpression of pancreatic TLR4 or TGF‐β1 which was paralleled by an increased number of TLR4‐positive or TGF‐β1‐positive Mφs or PSCs in ALC‐fed rats. *In vitro*, TLR4 or TGF‐β1 production in Mφs or RP‐2 cells was up‐regulated by LPS. LPS alone or in combination with TGF‐β1 significantly increased type I collagen and α‐SMA production and Smad2 and 3 phosphorylation in serum‐starved RP‐2 cells. TGF‐β pseudoreceptor BAMBI production was repressed by LPS, which was antagonized by Si‐TLR4 RNA or by inhibitors of MyD88/NF‐kB. Additionally, knockdown of Bambi with Si‐Bambi RNA significantly increased TGF‐β1 signalling in RP‐2 cells. These findings indicate that LPS increases TGF‐β1 production through paracrine and autocrine mechanisms and that LPS enhances TGF‐β1 signalling in PSCs by repressing BAMBI via TLR4/MyD88/NF‐kB activation.

## 1 INTRODUCTION

Pancreatic fibrosis is a key pathological feature of alcoholic chronic pancreatitis (ACP) in which pancreatic stellate cells (PSCs) play a critical role.^1^, ^2^, ^3^, ^4^ Quiescent PSCs are activated by ethanol, its metabolites and reactive oxidation species resulting in their conversion into myofibroblast‐like cells which express the cytoskeletal protein alpha‐smooth muscle actin (α‐SMA) and which have the capacity to proliferate, synthesize extracellular matrix proteins including type I collagen (Col1).^5^, ^6^ Many cellular and matricellular PSC proteins are involved in the activation process, including cytokines, cell‐surface receptors, signal transduction molecules and factors that regulate PSC gene expression at the transcriptional and post‐transcriptional levels.^1^, ^2^, ^3^ Transforming growth factor‐beta 1 (TGF‐β1) was found to regulate activation and proliferation of PSCs in an autocrine fashion via Smad2, Smad3 and ERK pathways.^4^, ^7^, ^8^ Therefore, activation of PSCs is a critical step in the development of pancreatic fibrosis. Additionally, an important role for macrophages (Mφs) as regulators of inflammation and fibrosis in mice and human chronic pancreatitis has recently become apparent.^9^

The fact that only 5%–10% of alcoholics eventually develop overt pancreatitis indicates that ethanol alone is insufficient to lead to clinical pancreatitis.^1^, ^4^, ^10^ Over the last decade, bacterial endotoxin lipopolysaccharide (LPS) has been recognized as a trigger of fibrogenesis and PSC activation during ACP.^11^ Toll‐like receptor 4 (TLR4) is the primary receptor implicated in signal transduction events induced by LPS.^12^ Recently, we demonstrated that LPS enhances the production of chemotactic cytokines and TGF‐β1 in human Mφs and that LPS binds to TLR4 on rat PSC to activate downstream effector NF‐kB through adaptor protein MyD88 and regulate cytokine expression.^13^, ^14^ However, the interplay between Mφs and PSCs as well as the molecular link between pancreatic inflammation and fibrogenesis in ACP remains unclear.

This study was designed to determine mechanisms of ACP by which LPS enhances TGF‐β1 signalling and pancreatic fibrosis using an *in vivo* model of chronic alcohol (ALC) feeding challenged with repeated LPS injection and *in vitro* Mφ and PSC models.

## 2 METHODS

### 2.1 *In vivo* model of chronic ALC exposure ± LPS injections

Twenty‐two male Sprague‐Dawley rats (120 ± 10 g) were fed with an ALC liquid diet (ALC; 15 g/kg/d; 36% of total calories) according to the general formulation of Lieber‐DeCarli 15 for 10 weeks. The rats were randomly divided into 2 groups on the 6th day of week 8: (1) ALC‐fed rats in which LPS (3 mg/kg body weight) were administered weekly via the tail vein injection for 3 weeks; or (2) control ALC‐fed rats that received a volume of saline identical to the volume of LPS solution. Eleven animals from each group were killed at the end of week 10. Each pancreas was retrieved and cut into 3 parts (proximal, middle and distal pancreas). Samples were further divided into smaller pieces and fixed in 10% neutral buffer formalin or snap‐frozen in liquid nitrogen. The study was performed in accordance with the guiding principles for the care and use of laboratory animals approved by the Ethics Committee on Animal Experiments of the First Hospital of Jilin University.

### 2.2 Patient's cohorts

A total of 29 ACP patients and 25 healthy controls were recruited at our Liver Unit from May 2008 through May 2016 in the first hospital of Jilin University. The diagnosis of ACP was based on a daily intake of alcohol of >150 g for at least 5 years or >60 g for at least 10 years and on a typical clinical history and on one or more of the following criteria: pancreatic calcifications, typical pancreatic histology, imaging findings on retrograde cholangiopancreatography, ultrasonography or computed photography, and steatorrhea.^16^ Serum LPS was measured using a Gram‐negative bacterial LPS detection kit according to the manufacturer's protocols (Gold Mountainriver, Beijing, China). Other aetiologies for chronic pancreatitis were excluded. Endotoxemia was defined as more than 2 times upper limits of normal healthy control. Serum TGF‐β1 was measured with ELISA kit (Uscn Life Science Inc, TX, USA). The study was approved by the hospital's Ethics Committee. All participants signed informed consent.

### 2.3 Generation and culture of rat Mφs and PSCs

To prepare monocyte‐derived Mφ, rat peripheral blood mononuclear cells (PBMCs) were isolated from buffy coat preparation using human lymphocyte separation medium (Dakewe, China), and then monocytes were further enriched by CD14 magnetic beads (Miltenyi Biotec). Monocytes were cultured in 6‐well culture plates or 4‐well Lab‐Tek^®^ chamber slides with complete RPMI medium containing 10 ng/mL human M‐CSF. Rat Mφs were used from Day 6 onwards.^17^ The immortalized rat PSC line, RP‐2, was cultured in DMEM supplemented with 25 mmol/L Herpes buffer and 10% FBS as described.^14^

### 2.4 Aniline blue collagen stain

Formalin‐fixed, paraffin‐embedded sections (5 μm) were dewaxed and rehydrated. The sections were stained with 0.7% ponceau red solution for 10 seconds, followed by sequential incubation in 0.2% glacial acetic acid and 1% phosphomolybdic acid hydrate solution. The sections were then stained with aniline blue solution (2 g aniline blue in 1% glacial acetic acid. The area of fibrotic (blue) regions per section was determined by computer image analysis system (Image Pro Plus 6.0). The percentage of fibrosis for each individual animal was calculated from 10 randomly selected high power fields (HPFs; ×400) per specimen.

### 2.5 Immunohistochemistry and Immunofluorescence

Formalin‐fixed, paraffin‐embedded pancreatic sections were dewaxed and rehydrated. RP‐2 cells or Mφs were cultured in 4‐well Lab‐Tek chamber slides for 24 hour in the presence or absence of LPS (100 ng/mL). The slides were fixed for 30 minutes in acetone at 20°C. Pancreatic sections or chamber slides were, respectively, incubated with mouse anti‐human α‐SMA monoclonal antibody (1:300) (Santa Cruz, USA) or rabbit anti‐BAMBI polyclonal antibody (1:300) (Protein Tech, USA) or rabbit anti‐TLR4 polyclonal antibody (1:100) (Boster, Wuhan, China) at room temperature for 1 hour, followed by biotinylated anti‐IgG and streptavidin‐peroxidase. Development of the chromogenic colour reaction was accomplished using 3‐amino‐9‐ethylcarbazole (Maixin, Fuzhou, China). To identify TLR4‐positive or TGF‐β1‐positive Mφs/PSCs in the pancreas, l section (4 μm) was incubated with primary antibodies against F4/80 (Santa Cruz, USA) or α‐SMA, and TLR4 (Boster, Wuhan, China) or TGF‐β1 (Santa Cruz, USA), followed by goat anti‐rabbit Alexa Fluor 555 (Life Technology, USA) or FITC labelled goat antimouse (Sigma, USA) secondary antibodies. Images were captured using an Olympus BX51 TRF Fluorescent/light microscope (Olympus, Tokyo, Japan). The number of α‐SMA‐positive PSCs or TLR4‐positive Mφs/PSCs or BAMBI‐positive PSCs or TGF‐β1‐positive Mφs/PSCs was determined by counting 10 randomly selected HPFs (×400) per specimen. The integrated optical density (IOD) of BAMBI‐positive PSC images was measured with computer image processing system (Image Proplus 6.0) by processing 10 randomly selected HPFs (×400) per specimen. The mean optical density (OD) per BAMBI‐positive PSC was counted.

### 2.6 Real‐time quantitative PCR(qPCR) analysis

RP‐2 cells were placed 6‐well culture plates and incubated in 0.5% FBS DMEM for 12 hour in the absence or presence of LPS (100 ng/mL) or TGF‐β1 (10 ng/mL), either individually or in combination. For inhibition experiments, the cells were pre‐treated with NF‐kB inhibitor Bay 11(10 μmol/L), MyD88 inhibitor ST2825 (10 μmol/L) for 30 minutes, or with small interfering TLR4 RNA (Si‐TLR4 RNA) or small interfere BAMBI RNA (Si‐BAMBI RNA) or small interfering control RNA for TLR4 or BAMBI, respectively, (Si‐Ctrl RNA) (Sangon Biotech, Shanghai, China) (2 μg + 2 μL FuGENE6) for 24 hour. Total RNA was then extracted from the treated cells using TRIzol reagent according to the manufacturer's instructions (Dingguo, Beijing, China). Levels of mRNA were determined by qRT‐PCR. Briefly, 1 μg RNA was reverse transcribed to cDNA using a PrimeScript^®^ RT reagent kit (Takara, Dalian, China). PCR analysis was performed using power SYBR Green PCR Master Mix (Life Technologies, Warrington, UK) with respective primer pairs on the Applied Biosystems 7500 Sequence Detection System. Data were normalized to β‐actin, and fold change in target gene expression converted to Ct values using the Delta‐Delta Ct method. qRT‐PCR assays were repeated three times.

### 2.7 Western blot

RP‐2 cells were cultured for 45 minutes or 12 hour or 24 hour in the absence or presence of stimulators, respectively. The cells were alternatively pre‐treated with Bay 11 or ST2825 for 30 minutes or Si‐RNA for 48 hour. Total cell protein or nuclear protein or cytoplasmic protein was extracted from the cells using, respectively, an Animal Cell Active Protein Extraction Kit, or a Nuclear and Cytoplasmic Protein Extraction Kit (Sangon Biotech, Shanghai, China). Protein samples (20 μg each) from RP‐2 cells or pancreatic tissue samples were separated on 10% SDS‐PAGE gels, and transferred to nitrocellulose. After washing with Tris buffer saline containing 0.1% Tween 20 (TBS/T) and blocking with 2.5% non‐fat milk, the membranes were separately incubated at 4°C overnight with rabbit polyclonal anti‐TLR4 (Boster, Wuhan, China), anti‐col1, anti‐BAMBI, anti‐α‐SMA (Protein Tech, USA), anti‐IkBα, anti‐pIkBα, anti‐p65, anti‐pSmad2, anti‐pSmad3, anti‐Smad1/2/3 antibodies, goat polyclonal anti‐p50 (Santa Cruz, USA), rabbit polyclonal anti‐pERK1/2, anti‐p‐p38 antibodies (Protein Tech, USA), rabbit polyclonal anti‐GAPDH antibody (Beyotime, Shanghai, China). Blots were incubated for 1 hour with HRP‐linked goat anti‐rabbit IgG or HRP‐labelled Donkey Anti‐Goat IgG (Beyotime) and washed extensively with TBS/T before detection using the ECL system (Thermo, USA).

### 2.8 Enzyme‐linked immunosorbent assay

The supernatants from homogenates of pancreatic samples or cultured Mφs and RP‐2 cells were measured with ELISA kits for Col1A1, or TGF‐β1 (Uscn Life Science Inc, TX, USA) according to the manufacturer's protocols. Briefly, microtitre wells were pre‐coated with 100 μL of each standard or supernatants for 1 hour at 37°C. The plates were then developed by sequential addition of biotinylated primary antibody, avidin‐conjugated horseradish peroxidase and tetramethylbenzidine substrate solution, and the colour reaction was measured at 450 nm.

### 2.9 Statistical analysis

GraphPad Prism (version 5.0, GraphPad Software Inc. San Diego, CA) was used to run all statistical tests. Data were presented as mean ± SEM. *P* < .05 was considered to be statistically significant.

## 3 RESULTS

### 3.1 LPS enhances pancreatic fibrosis and PSC activation in ALC‐exposed rats

**Figure 1:**
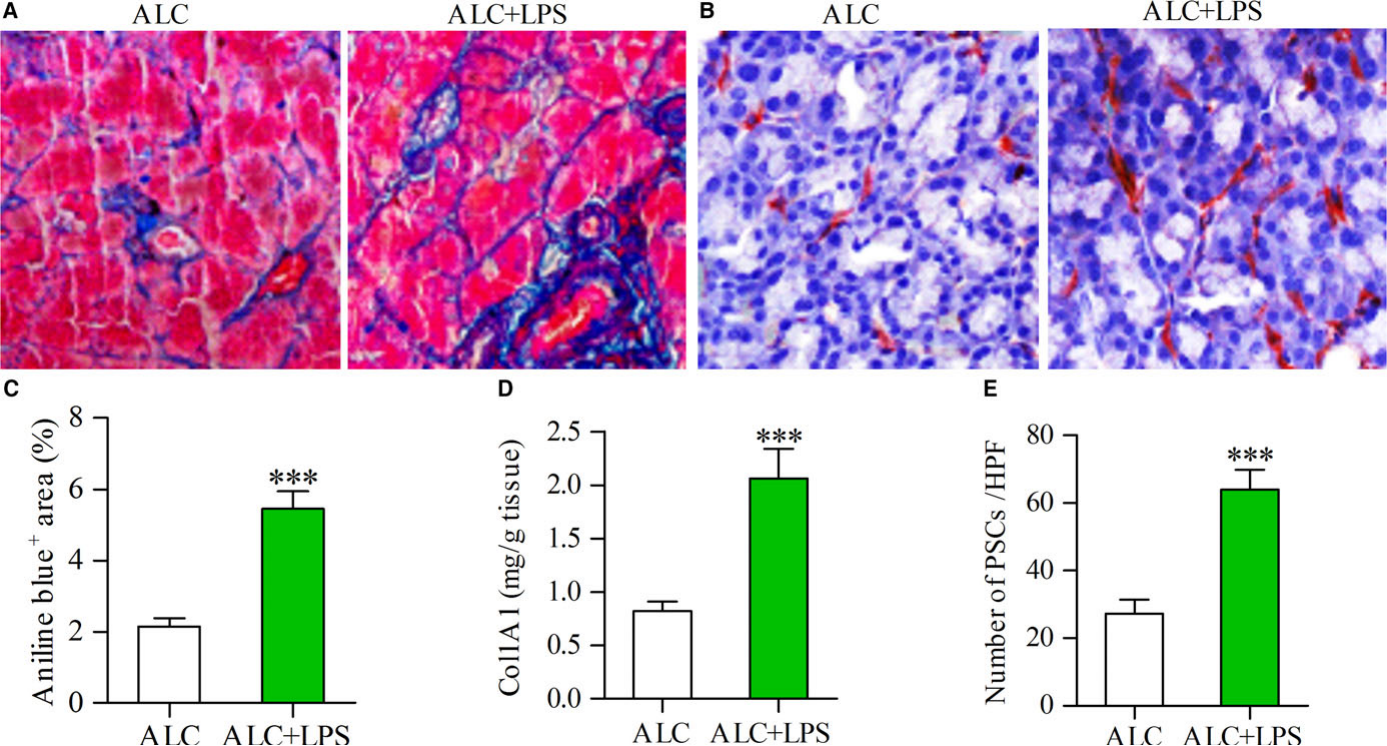
LPS was associated with increased expression of collagen and activation of PSCs. Aniline blue staining showing collagen deposition in the pancreas (blue) of alcohol (ALC, 15 g/kg/d)‐fed rat and ALC rat plus LPS (3 mg/kg once a week for 3 weeks) repeated injections (ALC + LPS) (A); Immunostaining showing the activated PSCs in the pancreas of ALC rat and ALC + LPS rat (B); The area of collagen deposition (C) or the content of Col1A1 (D) or the number of activated PSCs (E) in the pancreas of ALC + LPS rats was significantly more than that of ALC rats. Student's *t* test, ***P < .001 vs ALC rats (n = 11/group)

As pancreatic fibrosis is a key pathological feature of ACP in which PSCs play a critical role, we first measured collagen deposition and Col1A1 content in pancreatic tissue samples from ALC rats or ALC + LPS rats by aniline blue stain or ELISA, respectively. Repeated LPS injection increased collagen deposition in the pancreas of ALC rats (Figure 1A). The percentage of fibrotic (blue) regions and Col1A1 content were significantly higher in the pancreas of ALC + LPS rats than those of ALC rats (Figure 1C and D) (both *P* < .001). As expected, a few activated PSCs were found in ALC rats but many in ALC + LPS rats (Figure 1B). The number of activated PSCs was significantly increased in ALC + LPS rats vs ALC rats (Figure 1E) (*P* < .001).

### 3.2 LPS induces overexpression of pancreatic TLR4 in ALC‐exposed rats

**Figure 2:**
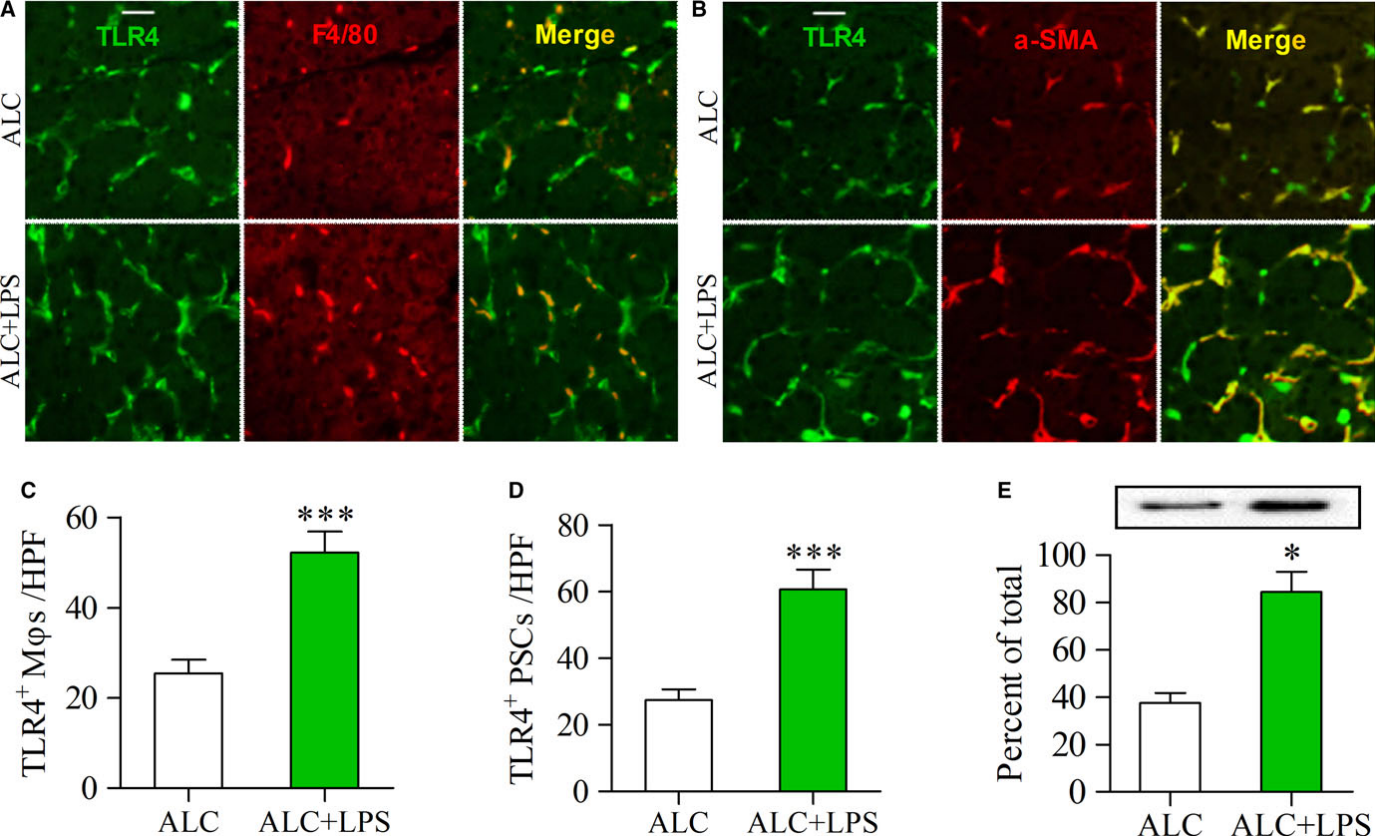
Enhanced expression of TLR4 in the pancreas of ALC‐fed rats with LPS injections. (A, B) Double‐immunofluorescence labelling using antibodies for TLR4 and F4/80 or α‐SMA. Yellow, colocalization of two antibodies. Scale bar = 50 μm; (A) Yellow staining indicates TLR4‐positive Mφs; (B) Yellow staining indicates TLR4‐positive PSCs. The number of TLR4‐positive Mφs/PSCs was higher in the pancreas of ALC + LPS rats than that of ALC rats (C, D); (E) The expression of TLR4 protein was examined by Western blot analysis (top panels) and the results, normalized with GAPDH, are the mean ± SEM of triplicate determinations (bottom panels). Student's *t* test, *P < .05, ***P < .001 vs ALC rats (n = 11/group)

To confirm whether TLR4 pathway was associated with the activation of PSCs and Mφs, we detected the expression of TLR4 protein in F4/80‐positive Mφs or α‐SMA‐positive PSCs in the pancreatic _tissue samples by double‐labelled immunofluorescence. A few TLR4‐positive Mφs or TLR4‐positive PSCs were found in ALC rats, but many of each cell type were present in ALC + LPS rats (Figure 2A and B). The number of TLR4‐positive Mφs/PSCs was significantly increased in ALC + LPS rats compared to ALC rats (Figure 2C and D) (both *P* < .001). Similarly, TLR4 protein in pancreatic tissues was also statistically increased in ACL + LPS rats vs ALC rats as assessed by Western blot analysis (Figure 2E) (*P* < .05).

### 3.3 LPS induces low expression of BAMBI in PSCs of ALC‐exposed rats

**Figure 3:**
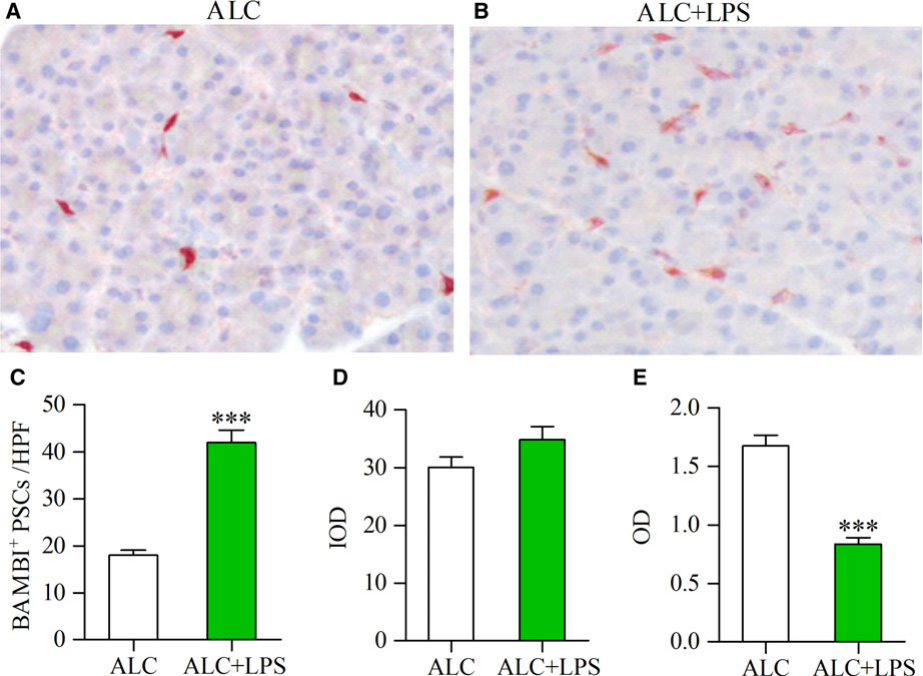
Decreased expression of BAMBI in the PSCs of ALC‐fed rats with LPS injections. Immunohistochemical staining showing strong positive staining for BAMB in several PSCs of ALC rat (A), but weak staining in more PSCs of ALC + LPS rat (red) (B). The number of BAMBI‐positive PSCs in the pancreas of ALC and ALC + LPS rats (C); Computer image analysis showed that the integrated optical density (IOD) of BAMBI‐positive PSCs in the pancreas of ALC and ALC + LPS rats (D); The mean optical density (OD) per BAMBI‐positive PSC was used for comparing the expression intensity of BAMBI in PSCs of ALC and ALC + LPS rats (E). Student's *t* test, ***P < .001 vs ALC rats (n = 11/group)

To confirm whether BAMBI was decreased in PSCs by LPS, we detected the expression of BAMBI protein by immunohistochemical staining. The BAMBI‐positive cells are located in periacinar and perilobular areas (Figure 3A and B), which were identified as PSCs using double‐labelled immunofluorescence for BAMBI and α‐SMA (data not shown). There is strong positive staining for BAMBI in several PSCs of ALC rat, but weak staining in more PSCs of ALC + LPS rat (Figure 3A and B). The number of BAMBI‐positive PSCs was significantly higher in ALC + LPS rats than that in ALC rats (Figure 3C) (*P* < .001); The IOD of BAMBI‐positive PSCs in the pancreas was not significant different between ALC and ALC + LPS rats, whereas the mean OD per BAMBI‐positive PSC was significantly decreased in ALC + LPS rats as compared to ALC rats (Figure 3D and E) (*P* < .001), indicating that LPS administration induces low expression of BAMBI in PSCs and promotes the cell proliferation in the pancreas of ALC‐fed rat model.

### 3.4 Enhanced expression of TGF‐β1 by LPS is positively correlated with Col1A1 expression in the pancreas after ALC exposure

**Figure 4:**
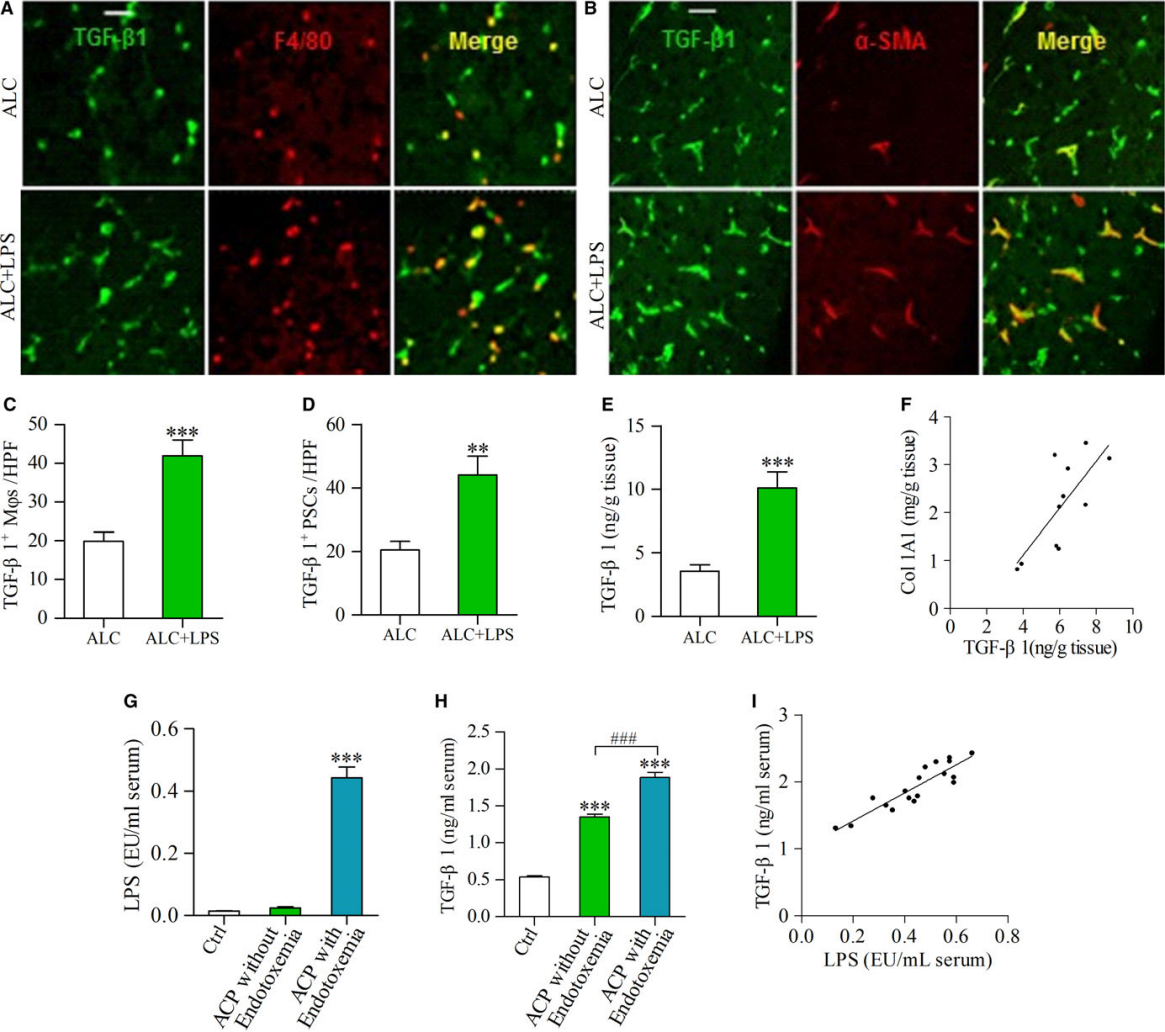
TGF‐β1 production was enhanced by LPS in the pancreas of ALC‐fed rats and in the sera of ACP patients. (A, B) Double‐immunofluorescence labelling using antibodies for TGF‐β1 and F4/80 or α‐SMA. Yellow, colocalization of two antibodies. Scale bar = 50 μm; (A) Yellow staining indicates TGF‐β1‐positive Mφs; (B) Yellow staining indicates TGF‐β1‐positive PSCs. The number of TGF‐β1‐positive Mφs/PSCs was higher in the pancreas of ALC + LPS rats than that of ALC rats (C, D); (E) The expression of TGF‐β1 protein was determined by ELISA. Student *t* test, **P < .01, ***P < .001 vs ALC rats. (F) There was a positive correlation between TGF‐β1 and Col1A1 in the pancreas of LPS‐treated ALC rats. Pearson's correlation test, *r* = .541, *P* < .01 (n = 11/group). (G) The LPS levels in the sera of ACP patients and healthy control (Ctrl); (H) The TGF‐β1 levels in the sera of ACP patients with/without endotoxemia and Ctrl group. One‐way ANOVA followed by Bonferroni post hoc test, ***P < .001 vs Ctrl, ^###^ *P* < .001 vs ACP without endotoxemia. (I) There was a positive correlation between TGF‐β1 and LPS in the sera of ACP patients with endotoxemia. Pearson's correlation test, *r =* .609, *P <* .01. (Ctrl: n = 25; ACP without endotoxemia: n = 11; ACP with endotoxemia: n = 18)

As TGF‐β1 is a major driver of pancreatic fibrosis, we measured the expression of TGF‐β1 protein in F4/80‐positive Mφs or α‐SMA‐positive PSCs in the pancreatic tissue samples by double‐labelling immunofluorescence. Only a few TGF‐β1‐positive Mφs or TGF‐β1‐positive PSCs were found in ALC rats, but many of these cells were present in ALC + LPS rats (Figure 4A and B). The number of TGF‐β1‐positive Mφs/PSCs was significantly increased in ALC + LPS rats vs ALC rats (Figure 4C and D) (*P* < .001, *P* < .01). Consistent with the immunofluorescence findings, ELISA analysis revealed that TGF‐β1 protein in pancreatic tissues was also significantly increased in ACL + LPS rats compared to ALC rats (Figure 4E) (*P* < .001). Additionally, enhanced production of TGF‐β1 was positively correlated with Col1A1 content in the pancreas of ALC + LPS rats (Figure 4F) (*r* = .541, *P* < .01).

### 3.5 Increased TGF‐β1 levels induced by LPS in the sera of ACP patients

Eleven ACP patients without endotoxemia were male, with mean age of 43.84 ± 8.62 years. Similarly, 94.4% (17 of 18) of ACP patients with endotoxemia were male, with a mean age of 44.16 ± 9.25 years. Of 25 healthy controls, 15 were male and 10 were female, with mean age of 29.9 ± 5.66 years. Of 29 ACP cases, 62% of cases had endotoxemia. The serum LPS values were significantly higher in ACP with endotoxemia than those in healthy controls (*P* < .001) (Figure 4G). The serum TGF‐β1 levels in ACP patients with/without endotoxemia were higher than those in healthy controls (both *P* < .001), with the highest level in ACP with endotoxemia (*P* < .001) (Figure 4H). There was a positive correlation between serum LPS and TGF‐β1 levels in ACP with endotoxemia (*r* = .607, *P* < .01) (Figure 4I).

### 3.6 LPS up‐regulates TLR4 and induces TGF‐β1 production in cultured Mφs and PSCs

**Figure 5:**
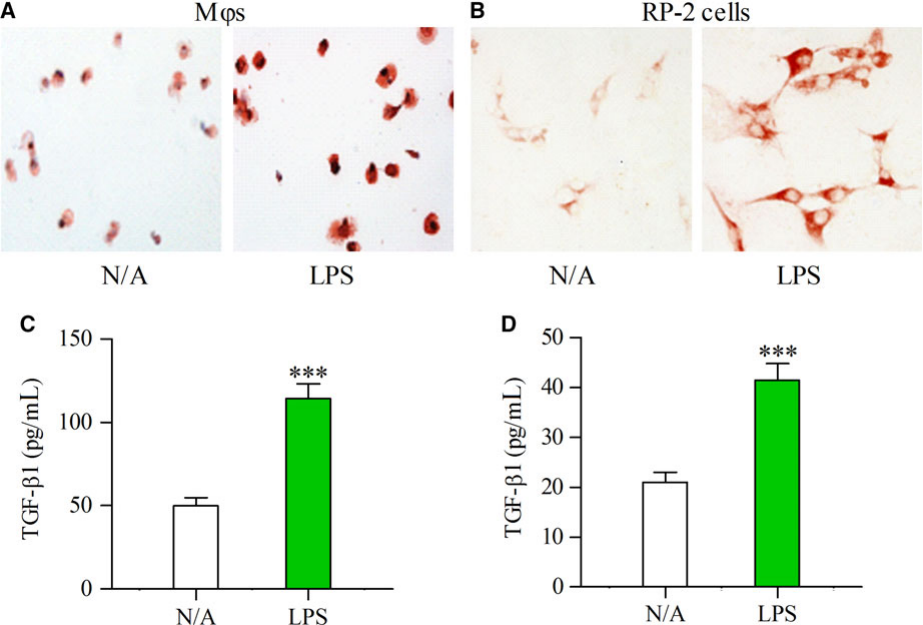
Expression of TLR4 and TGF‐β1 was enhanced by LPS in cultured Mφs and PSCs. Immunocytological staining showing the enhanced expression of TLR4 by LPS (100 ng/mL) in cultured Mφs (A) and RP‐2 cells (B) The concentrations of TGF‐β1 were determined by ELISA in the supernatant of cultured Mφs (C) or RP‐2 cells (D) that had been incubated for 24 h in the absence (N/A) or presence of LPS (100 ng/mL). Student's *t* test, ***P < .001 vs N/A (n = 6/group)

We next measured the production of TLR4 and TGF‐β1 in cultured Mφs and RP‐2 cells by immunocytochemistry or ELISA. The expression of TLR4 was enhanced in Mφs and RP‐2 cells under LPS stimulation (Figure 5A and B). Mφ and PSC are major producers of TGF‐β1 and active form of TGF‐β1 secreted by both cells only constitutes a small proportion of total TGF‐β1 (latent and active forms). The latent TGF‐β1 as a high molecular weight must be processed to a lower molecular weight, biologically active form which can be detected by commercial ELSA kit.^18^, ^19^ ELISA detection showed that the secretion of TGF‐β1 in cultured Mφs and RP‐2 cells was significantly increased under LPS stimulation compared to no addition (Figure 5C and D) (both *P* < .001).

### 3.7 LPS enhances TGF‐β1‐induced PSC activation and Col1 synthesis through down‐regulation of BAMBI

**Figure 6:**
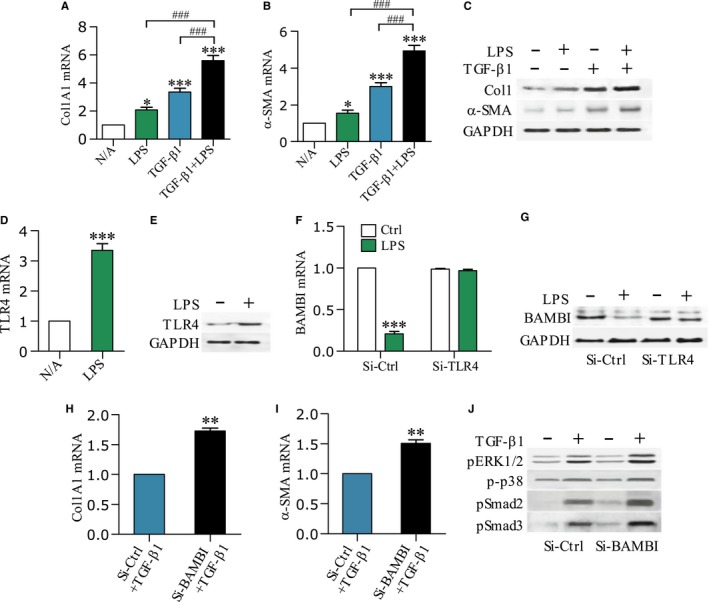
LPS sensitizes PSCs to TGF‐β1 and down‐regulates BAMBI. Col1A1 and α‐SMA mRNA levels (A, B) or Col1 and α‐SMA protein contents (C) in the RP‐2 cells after 12 h and 24 h of stimulation with LPS (100 ng/mL) or TGF‐β1 (10 ng/mL) or in combination were determined by qPCR or Western blot, respectively. One‐way ANOVA followed by Bonferroni post hoc test, *P < .05, ***P < .001 vs N/A; ^###^ *P* < .001 vs LPS or TGF‐β1 group. TLR4 mRNA levels (D) or protein contents (E) were determined in the RP‐2 cells that had been incubated for 12 h or 24 h in the absence or presence of LPS (100 ng/mL); BAMBI mRNA levels F or protein contents (G) were determined in the RP‐2 cells after 12 h or 24 h of stimulation with LPS (100 ng/mL); Before treatment, RP‐2 cells were pre‐transfected with either small interfere control RNA (Si‐Ctrl) or small interfere TLR4 RNA (Si‐TLR4) for 24 h; Col1A1 and α‐SMA mRNA levels (H, I) or ERK1/2 and p38 and Smad2/3 phosphorylations (J) were determined in the RP‐2 cells after 12 h or 24 h of stimulation with TGF‐β1 (10 ng/mL); Before treatment, RP‐2 cells were pre‐transfected with either Si‐Ctrl or small interfere BAMBI RNA (Si‐BAMBI) for 24 h. Student's *t* test, **P < .01, ***P < .001 vs Si‐Ctrl group (n = 6/group)

Having shown an effect of LPS on TGF‐β1 production after ALC exposure *in vivo* or in cultured Mφs and RP‐2, we next investigated the role of LPS or TGF‐β1 on PSC action. Col1A1 and α‐SMA mRNA expression levels were significantly increased in cultured RP‐2 cells under individual or combined treatment with LPS or TGF‐β1 as compared to non‐treated cells (*P* < .05, *P* < .001, *P* < .001, respectively), with the highest level of stimulation resulting from their combined effects (both *P* < .001) (Figure 6A and B). Similarly, Col1 and α‐SMA protein contents were also increased in cultured RP‐2 cells that were pre‐treated with LPS or TGF‐β1 or LPS + TGF‐β1 (Figure 6C).

To gain further insight into the mechanism of LPS on TGF‐β1 action, we next studied the production of TLR4 or BAMBI. TLR4 mRNA and protein were significantly increased in RP‐2 cells under LPS stimulation compared to no addition (Figure 6D and E) (*P* < .001). Notably, BAMBI mRNA and protein were decreased by LPS in RP‐2 cells that were pre‐transfected with Si‐Ctrl RNA, but they were unchanged by LPS in the cells pre‐transfected with Si‐TLR4 RNA (Figure 6F and G) (*P* < .001), suggesting that LPS‐mediated down‐regulation of BAMBI was TLR4‐dependent.

To further investigate the effect of BAMBI on TGF‐β1 action, we detected the TGF‐β1‐induced Col1A1 and a‐SMA mRNA expressions as well as TGF‐β1 activation of signalling pathways in the RP‐2 cells. The TGF‐β1‐induced Col1A1 and a‐SMA mRNA levels were significantly increased in the RP‐2 cells that were pre‐treated with Si‐BAMBI RNA as compared to Si‐Ctrl RNA‐treated cells (both *P* < .01) (Figure 6H and I). Similarly, TGF‐β1‐induced phosphorylations of ERK1/2 and p38 MAPK and Smad2/3 were also enhanced in the Si‐BAMBI RNA‐treated cells (Figure 6J).

### 3.8 LPS down‐regulates BAMBI through MyD88‐NF‐kB‐dependent pathway

**Figure 7:**
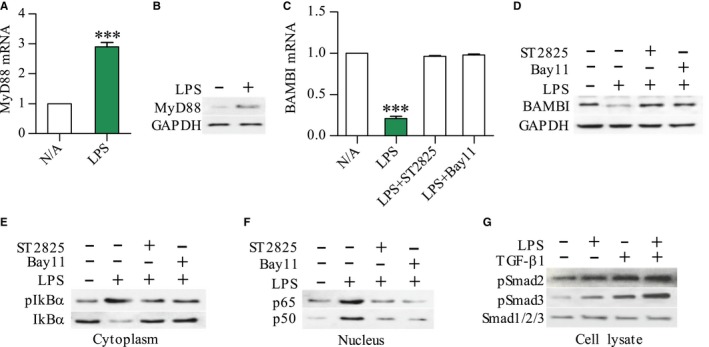
Down‐regulation of BAMBI by LPS through a MyD88/NF‐kB‐dependent pathway. MyD88 and BAMBI mRNA levels (A, C) or proteins (B, D) in the RP‐2 cells after 12 h and 24 h of stimulation with LPS (100 ng/mL) were determined by qPCR and Western blot, respectively; Western blot showing the expression of pIkBα or IkBα in cytoplasm and NF‐kB p65 or p50 in nucleus of RP‐2 cells after 45 min of LPS stimulation (E, F) For inhibition experiments, RP‐2 cells were pre‐treated for 30 min with NF‐kB inhibitor Bay11(10 μmol/L) or MyD88 inhibitor ST2825 (10 μmol/L); The expression of pSmad2 or pSmad3 or Smad123 in whole cell lysate of RP‐2 cells that had been stimulated with LPS (100 ng/mL) or TGF‐β1 (10 ng/mL) or in combination for 12 h (G). Student's *t* test, ***P < .001 vs N/A (n = 6/group)

To establish if down‐regulation of BAMBI by LPS was MyD88/NF‐kB‐dependent, we first determined MyD88 mRNA and protein by qPCR and Western blot, respectively. MyD88 mRNA and protein were significantly increased in LPS‐treated RP‐2 cells compared to untreated cells (*P* < .001) (Figure 7A and B). Additionally, LPS‐decreased BAMBI mRNA and protein were fully antagonized by pre‐treatment of RP‐2 cells with ST2825 (MyD88 inhibitor) or Bay11 (NF‐kB inhibitor) (Figure 7C and D). To further confirm the role of LPS on NF‐kB activation and Smad2/3 phosphorylation, cytoplasmic pIkBα or IkBα, nuclear NF‐kB p65 or p50, and pSmad2/3 or Smad123 in cell lysates were detected by Western blot individually. LPS induced phosphorylation of IkBα and translocation of NF‐kB p65 or p50 into the nucleus, both of which were blocked by pre‐treating RP‐2 cells with ST2825 or Bay11 (Figure 7E and F). On the other hand, LPS alone or in combination with TGF‐β1 showed a delayed effect in eliciting phosphorylation of Smad2 and 3 in serum‐starved RP‐2 cells (Figure 7G).

## 4 DISCUSSION

Although excessive ALC consumption is commonly associated with pancreatitis, ethanol alone does not appear to be sufficient to initiate clinical pancreatitis.^1^, ^4^ This notion is further supported by the fact that administration of ethanol alone to experimental animals does not cause pancreatitis.^11^, ^20^ Currently, it is widely believed that ethanol sensitizes the pancreas to injury and that additional factors are required for the manifestation of overt pancreatitis.^1^, ^10^, ^21^ Bacterial endotoxin LPS has been recognized as a trigger factor, as shown by our clinical data that enhanced chemotactic factors and TGF‐β1 are found in the sera of ACP patients with endotoxemia as well as by other experimental evidence that an LPS challenge in ALC‐fed rats leads to pancreatitis.^10^, ^11^ As chronic ALC consumption is known to increase gut permeability and decrease the phagocytic capacity of Kupffer cells, it is possible that an inability to detoxify circulating endotoxin may make some drinkers susceptible to overt disease.^22^, ^23^ These observations underscore the difficulty of establishing animal models of ACP by ALC feeding alone. Recently, our study showed that male Sprague‐Dawley rats challenged with repeated LPS injections (3 mg/kg body weight once a week for 3 weeks) revealed up‐regulation of TLR4 and increase of cytokines/chemokines (TNF‐α, IL‐1, IL‐6, MIP‐1 and Rantes) as compared to control rats but no significant difference in pancreatic fibrosis between two groups (data not shown). Other studies have suggested that inclusion of a “second‐hit” in ALC feeding animal models is more clinically relevant.^11^, ^24^ In support of this, and consistent with a previous report,^11^ we successfully established a rat model of ALC‐associated pancreatic fibrosis by chronic ALC feeding in conjunction with LPS exposure. Additionally, our results show that in ALC‐fed rats, LPS injection significantly enhances activation of Mφ and PSC which are believed to play a central role in triggering inflammation and disease progression.^4^

TLR4, the major receptor of LPS, is expressed with co‐receptor CD14 on the cell surface of LPS‐responsive cells such as monocytes, HSCs and fibroblasts.^25^, ^26^ In previous *in vitro* studies, we have shown that LPS up‐regulates TLR4 and CD14 in human Mφs and rat PSCs, both of which produce chemotactic cytokines (MCP‐1, MIP‐1α and Rantes), which are believed to induce PSC activation and Mφ chemostasis.^4^, ^13^, ^14^, ^27^ We have also demonstrated that gut‐derived endotoxin has the capacity to up‐regulate TLR4, chemotactic cytokines and TGF‐β1 in the pancreas of chronic ALC‐fed rats (data not shown). In the present studies, we provide several lines of evidence that Mφs and PSCs are the primary targets that drive pancreatic fibrogenesis in response to LPS. First, activated Mφs and PSCs express high levels of TLR4 and are highly response to LPS both *in vivo* and *in vitro*; Second, TGF‐β1 was overproduced in Mφs and PSCs *in vivo* and *in vitro*; Lastly, LPS sensitizes quiescent (serum‐starved) RP‐2 cells to TGF‐β1. Our results also indicate that LPS increases TGF‐β1 production through PSC autocrine and Mφ paracrine pathways. However, acinar cell and type 2 Mφ but not type 1 Mφ have been recognized as targets of LPS to produce TGF‐β1.^24^, ^28^ Hence, TGF‐β1 paracrine signalling needs to be further clarified.

TGF‐β1 is the major biological factor that promotes the biosynthesis of extracellular matrices such as collagen and the formation of pancreatic fibrosis by activating PSCs.^29^ Recently, our studies indicate that TGF‐β1 promotes PSC functions, including cell migration, production of Col1, MMP‐1 and MMP‐2, and inhibition of TIMP‐2.^14^ Upon TGF‐β1 stimulation, the TGF‐β type II receptor (TβRII) kinase phosphorylates TβRI whereupon Smad2 and Smad3 (R‐Smad) become phosphorylated and form heterodimers with Smad4 (Co‐Smad); the complexes then translocate into the nucleus, bind to DNA, and activate the transcription of target genes.^7^ Studies have revealed that TGF‐β1 stimulates PSC activation in a Smad2‐dependent fashion, while TGF‐β1/Smad3 transduction pathway transmits signals to promote collagen synthesis and PSC activation.^7^, ^30^ IL‐1β‐induced TGF‐β1 production and TGF‐β1 autoinduction in PSCs were regulated through ERK1/2‐dependent pathway.^7^, ^8^ LPS‐elicited Smad2 phosphorylation as well as the overexpression of Col1 and α‐SMA in HSC‐T6 cells was involved p38 MAPK signalling activation.^31^ In this study, our results demonstrated that LPS synergized with TGF‐β1 to increase Col1 and α‐SMA production and LPS also showed a delayed effect in eliciting Smad2 and 3 phosphorylation in serum‐starved RP‐2 cells. The latter of which were supported by recent evidence showing that enhanced phosphorylation of Smad2/3 in LX‐2 cells occurs 12 hour after LPS stimulation.^32^ Additionally, knockdown BAMBI with Si‐BAMBI RNA significantly increased the expression of Col1A1 and α‐SMA mRNAs and the phosphorylation of Smad2/3 and p38 MAPK and ERK1/2 in RP‐2 cells, indicating BAMBI as a novel target molecule through which LPS regulates PSC sensitivity to TGF‐β1.

BAMBI is a TGF‐β family type I receptor that lacks an intracellular kinase domain and which blocks signal transduction after stimulation with ligands such as TGF‐βs, BMP and activin.^33^, ^34^ Expression of BAMBI inhibits TGF‐β signalling and promotes tumour growth and metastasis in human gastric cancer.^35^ In contrast, down‐regulation of BAMBI in non‐small cell lung cancer enhances TGF‐β signalling and tumour invasion.^36^ BAMBI knockdown reduces the anti‐adipogenic effects of TGF‐β1 and Wnt3a.^37^ BAMBI inhibits skin fibrosis by suppressing TGF‐β1‐induced fibroblast cell proliferation and excessive accumulation Col1.^38^ BAMBI down‐regulation sensitizes HSCs to TGF‐β1 and facilitates hepatic fibrosis.^39^ Whereas, overexpression of BAMBI in the lung of idiopathic pulmonary fibrosis patients and in TGF‐β1‐treated small airway epithelial cells may play a role in the process of pulmonary fibrosis.^40^ In this study, we demonstrated that LPS enhanced TGF‐β1‐induced activation and Col1 synthesis in quiescent (serum‐starved) RP‐2 cells by suppressing BAMBI. Additionally, knockdown BAMBI with Si‐BAMBI RNA significantly increased TGF‐β1‐induced PSC function and signalling pathway. As BAMBI suppression by LPS was followed by up‐regulation of TLR4 and MyD88 and phosphorylation of NF‐kB p50/p65, all of which were antagonized by Si‐TLR4 RNA or by inhibitors of MyD88/NF‐kB, we propose that upon LPS recognition, TLR4 undergoes oligomerization and recruits its downstream adaptors through MyD88‐dependent pathway to activate IkBα. These events in turn activate NF‐kB and regulate the expression of BAMBI in RP‐2 cells. Further studies for silencing p50 or p65 will help to clarify the relationship between BAMBI and NF‐kB dimerization, their precise location in PSCs.

In conclusion, in an ALC‐fed rat model that involves LPS challenge, TGF‐β1 is produced via paracrine and autocrine pathways in, respectively, Mφ and PSC, leading to PSC activation and Col1 synthesis. In *in vitro* studies, we show that LPS synergizes with TGF‐β1 to facilitate activation and collagen synthesis in PSC via mechanisms that involve repression of BAMBI via TLR4/MyD88/NF‐kB signalling. Our results show that targeting of bacterial endotoxins, or their downstream mediators, may be a promising therapeutic strategy for ACP.

## COMPETING INTERESTS

The authors declare that they have no competing interests.
